# Editorial: *Aeromonas* spp.-transmission, pathogenesis, host-pathogen interaction, prevention and treatment

**DOI:** 10.3389/fmicb.2023.1320343

**Published:** 2023-11-02

**Authors:** Maoda Pang

**Affiliations:** ^1^College of Veterinary Medicine, Nanjing Agricultural University, Nanjing, China; ^2^Jiangsu Key Laboratory for Food Quality and Safety-State Key Laboratory Cultivation Base of MOST, Institute of Food Safety and Nutrition, Jiangsu Academy of Agricultural Sciences, Nanjing, China

**Keywords:** *Aeromonas* spp., epidemiology, pathogenesis, resistance mechanism, treatment

Bacteria of the genus *Aeromonas* are ubiquitous in terrestrial environments and in close association with humans and animals. *Aeromonas* was originally known to cause infections in fish and other cold-blooded animals. This bacterium also widely targets immunocompromised animals and human hosts, resulting in wound infections, cellulitis, septicemia, and urinary tract infections. Currently, there are 36 species of *Aeromonas* and *Aeromonas hydrophila, Aeromonas dhakensis, Aeromonas veronii, Aeromonas salmonicida*, and *Aeromonas caviae* all cause significant economic losses in the aquaculture industry worldwide.

The continuous and ongoing efforts to understand *Aeromonas* are critical to drive the scientific discovery of preventative and treatment strategies for these infections. In this context, this Research Topic aimed to collect recent studies on the following themes: (1) mode of transmission between hosts; (2) pathogenesis and resistance mechanisms developed by this species; (3) host–pathogen interactions and immune evasion mechanisms; (4) prevention strategies to prevent infections; and (5) novel treatment strategies against the infection. This Research Topic is comprised of five original research articles from China, Norway, Denmark and Germany, which were contributed by 30 authors. These studies mainly focused on epidemiology, pathogenesis, resistance mechanisms and potential new treatments.

The most common bacterial disease in striped catfish in Vietnam is *Aeromonas* septicemia. Erickson et al. confirmed that the dominant species causing outbreaks of *Aeromonas* septicemia in striped catfish fingerlings in the Mekong Delta were *A. dhakensis* ST656 and *A. hydrophila* ST251. In this study, 30 *A. hydrophila* isolates characterized by PCR were further identified as *A. hydrophila* (5/30) and *A. dhakensis* (25/30) by whole-genome sequencing. This demonstrated that *A. dhakensis* is not a rare cause of *Aeromonas* septicemia, and the true burden of *A. dhakensis* has been masked due to the lack of diagnostic methods that can differentiate *A. dhakensis* from *A. hydrophila*. This study also showed that the immunogenic potential of outer membrane proteins and lipopolysaccharide regions was not significantly different between *A. dhakensis* and *A. hydrophila*, but the efficiency and effectiveness of current vaccines in preventing their infection should be determined.

Multidrug-resistant *Aeromonas* isolates that are circulating in aquatic environments and food production chains may spread antimicrobial resistance (AMR) to humans through food. Lee et al. investigated AMR and virulence factors of 22 *Aeromonas* strains isolated from ready-to-eat seafood, which were identified as *A. hydrophila, A. dhakensis, A. caviae, A. salmonicida, Aeromonas rivipollensis, Aeromonas media, Aeromonas piscicola*, and *Aeromonas bestiarum*. The results showed that the virulence factors responsible for adhesion and motility (Msh type IV pili, tap type IV pili, and polar flagella), the type II secretion system, and hemolysin were found in all strains. Furthermore, multiple AMR genes encoding class B, C, and D β-lactamases were present in all strains, and the distribution was species-related. In addition, other AMR genes located in mobile genetic elements, such as an IncQ-type plasmid, could disseminate AMR genes. This study demonstrated that *Aeromonas* strains circulating in the food chain may serve as vectors for spreading AMR genes to other bacteria that reside in the same environment.

Benzalkonium chloride (BAC) is one of the most commonly used disinfectants, and the excessive emissions of BAC in aquatic systems can trigger a variety of physiological responses in environmental microorganisms. Chacón et al. conducted the first study addressing resistance to BAC in an environmental *A. hydrophila* strain. This study isolated an *A. hydrophila* strain, INISA09, which was less sensitive to BAC, from a wastewater treatment plant in Costa Rica. A total of 15,762 missense mutations, which were mainly related to transport, antimicrobial resistance, and outer membrane proteins, were identified in strain INISA09. Quantitative proteomic analysis showed that when the strain was exposed to BAC, a significant upregulation of the expression of several efflux pumps and the downregulation of porin expression were found. Thus, the low susceptibility of strain INISA09 to BAC could be related to changes in the expression of genes and proteins related to the outer membrane, transmembrane transport, and fatty acid synthesis metabolic pathways. This study contributes to our understanding of how bacteria adapt to biocide contamination.

The iron uptake regulator (Fur) is a transcription factor that regulates genes related to iron homeostasis and pathogenicity; this regulator can also directly inhibit the expression of the type VI secretion system (T6SS) in bacteria such as *Edwardsiella tarda*. Li et al. investigated the function of Fur in *A. hydrophila*. Fur was found to activate the T6SS by directly binding to the *vipA* promoter in the T6SS gene cluster. In addition, inactivation of Fur resulted in significant defects in interbacterial competitive activity and pathogenicity both *in vitro* and *in vivo*. This study provides direct evidence that Fur positively regulates the expression and functional activity of the T6SS in *A. hydrophila*.

Chinese herbal medicines have been considered “green drugs” to replace chemotherapeutics and antibiotics in aquaculture. Guo et al. prepared a novel herbal extract combination of GF-7 composed of Galla Chinensis and Manganese Shell extracts, as well as the effective parts of Pomegranate peel and Scutellaria baicalensis Georgi extracts, which showed highly efficient antibacterial activity against various aquatic pathogens, such as *A. hydrophila, in vitro*. The treatment effect on *Micropterus salmoide* showed that the expression of the immune regulatory factors IL-1β, TNF-α, and Myd88 in the liver was regulated to varying degrees at different times. Moreover, GF-7 significantly reduced the mortality of *Micropterus salmoide* and reduced the degree of liver lesions. These findings highlighted the novel combination GF-7 as a potential natural medicine to prevent and treat aquatic bacterial diseases.

In summary, the studies have expanded our understanding of *Aeromonas*, especially in the epidemiology of *Aeromonas* in Vietnam, the antimicrobial resistance of *Aeromonas* isolated from ready-to-eat seafood, the mechanisms of low BAC susceptibility in *A. hydrophila*, the role of Fur in *A. hydropihla* infection, and the effect of herbal extracts on *A. hydrophila* infection.

## Author contributions

MP: Writing—original draft, Writing—review & editing.

